# Pre-deployment dissociation and personality as risk factors for post-deployment post-traumatic stress disorder in Danish soldiers deployed to Afghanistan

**DOI:** 10.1080/20008198.2018.1443672

**Published:** 2018-03-09

**Authors:** Beatriz Ponce de León, Søren Andersen, Karen-Inge Karstoft, Ask Elklit

**Affiliations:** a Danish National Centre of Psychotraumatology, Department of Psychology, University of Southern Denmark, Odense, Denmark; b Psychiatry, Afdeling for Traume- og Torturoverlevere, Region of Southern Denmark,Vejle, Denmark; c Research and Knowledge Centre, The Danish Veteran Centre, Ringsted, Denmark

**Keywords:** Dissociation, combat, PTSD trajectories, personality, Big Five model, neuroticism, • The study investigated the influence of pre-deployment dissociation in the development of PTSD 2.5 years after homecoming by using previously identified PTSD trajectories. • There were significant differences in pre-deployment dissociation levels for the six trajectories of PTSD symptoms. • Two unique groups of trajectories based on differences in pre-deployment dissociation were identified. • Previous history of trauma, pre-deployment dissociation and pre-deployment neuroticism were significant independent predictors of post-deployment PTSD. • The study emphasizes the multiplicity of factors involved in the development of PTSD.

## Abstract

**Objective**: This study investigated whether pre-deployment dissociation was associated with previously identified post-traumatic stress disorder (PTSD) symptom trajectories from before to 2.5 years after military deployment. Furthermore, it examined whether the tendency to dissociate, pre-deployment personality factors, conceptualized by the Big Five model, and previous trauma represented independent risk factors for post-deployment PTSD symptoms.

**Method**: This prospective study included the entire team of 743 soldiers from the Danish Contingent of the International Security Assistance Force 7 deployed to Afghanistan in 2009. Data consisted of self-report measures and were collected six times: before deployment; during deployment; and 1–3 weeks, 2 months, 7 months and 2.5 years after homecoming.

**Results**: The findings indicate significant associations between pre-deployment dissociation and six PTSD trajectories (*p *< 0.001, *η*^2^ = 0.120). Based on mean differences in dissociation for the six trajectories, two main groups emerged: a group with high dissociation scores at pre-deployment, which had moderate PTSD symptom levels at pre-deployment and fluctuated over time; and a group with low dissociation scores at pre-deployment, which had low initial PTSD symptom levels and diverged over time. Our study also confirmed previous findings of a positive association between neuroticism and dissociation (*r* = 0.31, *p* < 0.001). This suggests that negative emotionality may be a vulnerability that enhances dissociative experiences, although a causal link cannot be concluded from the findings. Finally, pre-deployment dissociation, pre-deployment neuroticism and a history of traumatic events, as independent factors, were significant predictors of post-deployment PTSD (*p* < 0.001, *R*^2^ = 0.158).

**Conclusions**: The study emphasizes the multiplicity of factors involved in the development of PTSD, and group differences in dissociative symptoms support the heterogeneity in PTSD. Further, this study points to specific aspects of personality that may be targeted in a clinical setting and in pre-deployment assessments in the military.

## La disociación y la personalidad antes del despliegue militar como factores de riesgo para el trastorno por estrés postraumático (TEPT) posterior al despliegue en soldados daneses destinados en Afganistán

**Objetivo**: En este estudio investigamos si la disociación previa a un despliegue militar estaba asociada con trayectorias de síntomas de TEPT previamente identificadas desde antes hasta 2,5 años después del despliegue militar. Además, examinamos si la tendencia a disociarse, los factores de personalidad previos al despliegue, conceptualizados por el modelo Big Five y el trauma previo, representaban factores de riesgo independientes para los síntomas de TEPT posteriores al despliegue.

**Método**: El estudio prospectivo actual incluyó a todo el equipo de 743 soldados del contingente danés de la Fuerza de Asistencia Internacional a la Seguridad Internacional 7 desplegados en Afganistán en 2009. Los datos consistieron en medidas de autoinforme, y se recogieron 6 veces: antes del despliegue, durante el despliegue. 1–3 semanas después del regreso al hogar, 2 meses después del regreso al hogar, 7 meses después del regreso al hogar y 2.5 años después del regreso al hogar.

**Resultados**: Los hallazgos indican asociaciones significativas entre la disociación previa al despliegue y seis trayectorias de TEPT (*p* < 0.001, *η*^2^ = 0.120), y en base a diferencias medias en la disociación para las seis trayectorias surgieron dos grupos principales: Un grupo con puntuaciones de disociación altas previas al despliegue, que tenían niveles moderados de síntomas de TEPT antes del despliegue y fluctuaban con el tiempo. Un segundo grupo con puntuaciones de disociación bajas previas al despliegue, que tenían bajos niveles iniciales de síntomas de TEPT y divergían con el tiempo. Además, nuestro estudio confirmó los hallazgos previos sobre una asociación positiva entre el neuroticismo y la disociación (r = 0.31, *p* < 0.001). Esto podría sugerir que la emocionalidad negativa puede ser una vulnerabilidad que aumenta las experiencias disociativas, aunque no se puede concluir un vínculo causal a partir de los hallazgos. Finalmente, la disociación previa al despliegue, el neuroticismo previo al despliegue y una historia de eventos traumáticos, como factores independientes, fueron predictores significativos de TEPT posterior al despliegue (*p* < 0.001, *R*^2^ = 0.158).

**Conclusiones**: El estudio enfatiza la multiplicidad de factores involucrados en el desarrollo del TEPT y las diferencias grupales en los síntomas disociativos apoyan la heterogeneidad en el TEPT. Además, este estudio apunta a aspectos específicos de la personalidad en los que se puede trabajarse en un entorno clínico, así como en las evaluaciones previas al despliegue en el ejército.

## 派往阿富汗的丹麦士兵中，入伍前分离和人格是服役后创伤后应激障碍（PTSD）的风险因子

**目标：**在本研究中我们考察了入伍前的分离症状是否和从入伍前到入伍2.5年后的PTSD 症状轨迹相关联。进一步，我们考察了这种分离倾向性、入伍前的人格因子（大五模型）和前期创伤是否代表了不同的入伍后 PTSD风险因子。

**方法：** 本前瞻研究包括2009年阿富汗国际维和部队丹麦分遣队的全部743名士兵，数据包括在6个时间点收集的自评测量：入伍前、服役中，复员1-3周后、2个月、7个月和2.5年后。

**结果：**研究的发现指出入伍前分离和六个 PTSD 轨迹的显著关联 (*p* < 0.001, *η*^2^ = 0.120)，根据六个轨迹的分离症状平均数差异，出现了两个主要分组：一个组入伍前高分离，同时有中等的 PTSD 症状，并随着时间波动；第二个组在入伍前低分离，并有较低的 PTSD 症状水平但随着时间出现分支。另外，我们的研究验证了前人发现，说明神经质和分离之间的正相关 (r = 0.31, *p* < 0.001)。这可能提示负面情绪可能是增加分离体验的易感因素，尽管还不能从这些发现出总结出因果链。最后，作为独立因素的入伍前分离、入伍前神经质和创伤事件历史，都是服役后 PTSD 的显著预测指标。

**结论：**本研究强调了在 PTSD 发展中多种因子，还有分离症状支持 PTSD 的异质性的组间差异。本研究更进一步指出在临床设置和军队入伍前评估中可能需要关注一些独特的人格特质。

## 1. Introduction

Combat exposure can lead to severe and pervasive psychological, physical and interpersonal problems, as manifested in post-traumatic stress disorder (PTSD) (Sheffler, Rushing, Stanley, & Sachs-Ericsson, 2016; Xue et al., 2015). However, combat exposure alone is not sufficient to cause the development of PTSD (Dohrenwend, Yager, Wall, & Adams, 2013; Richardson, Frueh, & Acierno, 2010). In the context of military assessment and pre-deployment screening, it is therefore essential to identify pre-existing vulnerabilities that may predict the individual risk of developing PTSD after military deployment. In recent years, research has highlighted the potential role of personality and behavioural predispositions as such pre-existing vulnerability factors for PTSD (Engelhard & van den Hout, 2007; JakšI, Brajkovi, Ivez, Topi, & Jakovljevi, 2012; Weinberg & Gil, 2016). One such potentially pre-existing vulnerability is dissociation (McCaslin et al., 2008; Murray, Ehlers, & Mayou, 2002).

Dissociation is defined by Bernstein and Putnam (1986) as ‘A lack of the normal integration of thoughts, feelings, and experiences into the stream of consciousness and memory’. According to Bernstein and Putnam, dissociation occurs to some degree in normal subjects, but there is high prevalence of dissociative symptoms among individuals with psychiatric disorders. In the context of combat-related PTSD, it has been found that the level of dissociation is significantly higher among combat-exposed individuals with PTSD compared to combat-exposed individuals without PTSD as well as healthy individuals with no combat exposure (Ozdemir, Celik, & Oznur, 2015). However, this was a cross-sectional study, and pre-trauma dissociation and life adversities prior to combat were not assessed. Thus, there is a need for prospective, longitudinal studies to examine whether dissociation is in fact a predisposing factor that increases the risk of PTSD.

The most characteristic indicators of dissociation involve negative symptoms such as memory loss or partial amnesia, loss of motor control and loss of somatosensory awareness (e.g. depersonalization) (Van Der Hart, Nijenhuis, Steele, & Brown, 2004), as well as depersonalization and derealization (Bernstein & Putnam, 1986; Spiegel & Cardeña, 1991), which are common in many psychiatric disorders. Positive symptoms such as intrusion of traumatic memories and somatoform dissociative symptoms have also been identified (Van Der Hart et al., 2004). Although intrusive dissociative experiences are core features of PTSD, empirical support has led to the addition of a dissociative subtype of PTSD as part of the PTSD diagnosis in the *Diagnostic and Statistical Manual of Mental Disorders* (5th ed.; DSM-5) (American Psychiatric Association, 2013). For instance, recent studies of veterans and civilian PTSD populations have identified a subgroup with elevated dissociation scores with an emphasis on depersonalization and derealization symptoms (Steuwe, Lanius, & Frewen, 2012; Tsai, Armour, Southwick, & Pietrzak, 2015; Wolf et al., 2012a, 2012b).

However, the relationship between dissociation and PTSD is complex, and there is still much debate about the classification of the dissociative PTSD subtype (Dorahy & Van Der Hart, 2015). Dissociative symptoms may not only be an integral part of PTSD symptomatology (Dalenberg & Carlson, 2012), they may also be independent mechanisms for coping with overwhelming emotions (Kumpula, Orcutt, Bardeen, & Varkovitzky, 2011). This is consistent with this notion of peritraumatic dissociation, which is conceptualized as a coping mechanism (Kumpula et al., 2011).

Several studies have reported the influence of peritraumatic dissociation in the development of PTSD, e.g. in civilian populations exposed to mass shootings (Kumpula et al., 2011) or motor vehicle accidents (Ehlers, Mayou, & Bryant, 1998) and in combat veterans (Bremner et al., 1992; Marmar et al., 1994). However, a prospective longitudinal study showed that peritraumatic dissociation was no longer predictive of PTSD symptom severity at follow-up after controlling for PTSD symptom severity, measured within days of exposure (Marshall & Schell, 2002). Further, persistent dissociative experiences after the traumatic events rather than peritraumatic dissociation was associated with maladaptive behaviours, PTSD and acute stress disorder (Briere, Scott, & Weathers, 2005; Panasetis & Bryant, 2003). Moreover, many of the studies on peritraumatic dissociation relied on retrospective reports and are limited by the lack of specific temporal parameters for the occurrence of peritraumatic dissociation, warranting caution in the interpretation of results (Candel & Merckelbach, 2004; van der Hart, van Ochten, van Son, Steele, & Lensvelt-Mulders, 2008).

A further review of the evidence (Bryant, 2007) emphasizes that peritraumatic dissociation may not be the best predictor of PTSD, owing to its lack of sensitivity and specificity in predicting those at risk of PTSD; hence, other potential factors that may explain the link between dissociation and PTSD should be explored (Bryant, 2007). For instance, the predisposition to dissociate can occur as a result of sensitization by previous trauma (Dimoulas et al., 2007; Morgan et al., 2001; Morgan, Southwick, Hazlett, & Steffian, 2007), and personality traits can significantly influence the stress process and function as pre-existing vulnerability factors for PTSD (Engelhard & van den Hout, 2007; JakšI et al., 2012; Weinberg & Gil, 2016).

To this end, studies aiming to clarify the link between dissociation and personality constructs from the Five-factor model revealed a positive correlation between neuroticism and dissociation (Kwapil, Wrobel, & Pope, 2002; Spindler & Elklit, 2003). Nevertheless, these studies used a cross-sectional design, and causal or directional inferences cannot be established. Hence, the personality trait neuroticism and dissociation is of particular interest in the context of combat-related PTSD.

Individuals with high neuroticism scores tend to appraise situations (even banal events) as highly threatening (Vollrath, 2001). Neuroticism is a general indicator of a vulnerability to experience anxiety and sensitivity to stress (McCrae, 2010), and has been linked to emotional disengagement and avoidant coping (Carver & Connor-Smith, 2010). Thus, albeit speculative, it is possible that neuroticism enhances dissociative tendencies, hence increasing the risk of trauma-related psychopathology. It is therefore important to explore the relationship between neuroticism and dissociation and the combined effect of these factors, as well as the specific trait contributors in the development of combat-related PTSD. Moreover, research on the influence of pre-trauma dissociation is limited. Many studies have been conducted retrospectively or in military training settings, and the lack of longitudinal studies renders the findings inconclusive.

To our knowledge, this is the first prospective longitudinal study to examine whether pre-deployment dissociation is associated with the risk of PTSD 3 years after deployment (2.5 years after homecoming). In a previous study based on the same data set of Danish soldiers deployed to Afghanistan in 2009 (the USPER study; for details see Andersen, Karstoft, Bertelsen, & Madsen, 2014; Berntsen et al., 2012), six trajectories of PTSD symptoms were identified (Andersen et al., 2014). These trajectories consisted of a low–stable or resilient group with low PTSD symptom levels across all measurements (78.1% of the sample) and five other groups which had fluctuating symptom levels across the measurements. Owing to the assumed risk of post-deployment PTSD associated with pre-deployment dissociation, we would expect high dissociation levels in the five trajectories with fluctuating symptom levels.

In the current study, we examine whether pre-deployment dissociation is associated with the six trajectories from the USPER cohort study. Furthermore, we estimate the interaction effect between neuroticism and dissociation in the development of PTSD, and assess whether personality factors and the tendency to dissociate may represent independent risk factors for PTSD symptoms (Briere et al., 2005; McCaslin et al., 2008; Murray et al., 2002). We thus explore the influence of the Big Five personality traits (Costa & McCrae, 1992): extraversion, agreeableness, conscientiousness, neuroticism and openness to experience, measured before deployment, on pre-deployment dissociation and post-deployment PTSD.

Hence, in the current study we aim to investigate the following:

1. whether pre-deployment dissociation is associated with six pre-established PTSD trajectories
2. whether the five groups with fluctuating symptom levels exhibit high dissociation levels at pre-deployment
3. whether there is a relationship between pre-deployment dissociation, the Big Five personality traits and previous history of trauma
4. whether personality and pre-deployment dissociation represent risk factors for post-deployment PTSD symptoms measured by the PTSD Checklist – Civilian version (PCL-C) 2.5 years after homecoming.

## 2. Method

### 2.1. Participants

The current study is part of the prospective, longitudinal USPER study. The study included the entire team of 743 soldiers from the Danish Contingent of the International Security Assistance Force 7 deployed to Afghanistan in 2009. Of the total population (*N* = 743), 602 provided pre-deployment data, 37 of whom did not eventually deploy and three who died during deployment, and one had outlying PCL scores (> 3 *SD* above the mean at all time points). Hence, the study sample consisted of 561 soldiers. Of the total, 95% were male and 5% female. The age of the soldiers ranged from 18 to 57 years (*M* = 26.13, *SD* = 7.12). A total of 45.4% of the participants were single, 19.3% had a partner, 33.3% were married and 2% were divorced or separated, while 70.1% had primary and upper secondary school level education, and 29.8% had higher education. The years of service in the military before deployment ranged from under 1 year to 40 years (*M* = 5.48, *SD* = 6.90). The percentage of high-level PTSD symptoms (scores > 43) in the sample was 10% at the 2.5 year assessment.

### 2.2. Measures

#### 2.2.1. Dissociative Experience Scale (DES)

The DES (Bernstein & Putnam, 1986) is a 28-item self-report questionnaire that measures the frequency of dissociative experiences in both normal and clinical populations. The DES has been proven to be a useful and stable instrument in the assessment of dissociative experiences (Bernstein & Putnam, 1986; Dubester & Braun, 1995). Subjects are asked to rate the percentage of time (ranging from 0% to 100%) they have experienced each symptom in daily life. The total score is the mean of all items ranging from 0 to 100. The DES has good test–retest reliability of 0.84, with a Cronbach’s alpha of 0.93 for normal subjects (Frischholz et al., 1990). In the current sample, the DES showed a Cronbach’s alpha coefficient of 0.94.

#### 2.2.2. NEO Personality Inventory – Revised (NEO-PI-R)

The NEO-PI-R (Costa & McCrae, 1992) is a self-report instrument that measures five domains of personality: neuroticism, extraversion, openness to experience, agreeableness and conscientiousness. Each main domain consists of six facets. The scale contains 60 items which are scored on a five-point Likert scale from ‘strongly agree’ to ‘strongly disagree’. In a 6 year longitudinal study, the NEO-PI-R has shown satisfactory test–retest reliability and 6 year stability coefficients ranging from 0.63 to 0.83 (Costa & McCrae, 1988). Furthermore, it has high internal validity, Cronbach’s alpha for the domain scales ranges from 0.86 to 0.92 and internal consistency for the facet scales ranges from 0.56 to 0.81 (Costa, McCrae, & Dye, 1991). In this sample, the NEO-PI-R showed a Cronbach’s alpha coefficient for neuroticism of 0.79, extraversion 0.79, openness 0.70, agreeableness 0.74 and conscientiousness 0.84.

#### 2.2.3. PTSD Checklist – Civilian version (PCL-C)

The PCL-C (Blanchard, Jones-Alexander, Buckley, & Forneris, 1996) assesses the 17 symptoms of PTSD from the *Diagnostic and Statistical Manual of Mental Disorders* (4th ed.; DSM-IV) (American Psychiatric Association, 1994). The PCL has shown good reliability and validity with a variety of samples including combat veterans. The PCL has been validated in this sample of Danish soldiers against the Structured Clinical Interview for Axis I disorders of the DSM-IV (SCID-I) (First, Spitzer, Gibbon, & Williams, 2002; Karstoft, Andersen, Bertelsen, & Madsen, 2014). In this sample it showed high internal consistency (*α* = 0.94) (Andersen et al., 2014). The civilian version of the PCL was used to allow the respondents to report PTSD symptoms related to any traumatic events and not only symptoms caused by military experiences.

### 2.3. Other measurements

Additional pre-deployment variables included demographics, prior exposure to trauma measured by the Traumatic Life Event Questionnaire (TLEQ) (Kubany et al., 2000), which is a 21-item questionnaire that assesses a wide range of traumatic events including natural disasters, exposure to warfare, death of loved ones, armed robbery, and physical and sexual abuse. Scoring ranges from ‘never’ to ‘more than five times’, and whether fear, helplessness or horror was present (yes/no). The total score for the TLEQ was used. Measurements 1–3 weeks after homecoming included the Danger–Injury Exposure Scale, developed by the Danish military for screening purposes and included in previous studies (Berntsen et al., 2012). Cronbach’s alpha coefficient for the Danger–Injury Exposure Scale is 0.85. It consists of 10 items and assesses perceived war-zone stress. It includes the items: (1) being threatened by a weapon, (2) experiencing shooting, (3) being in areas with roadside bombs, (4) passing areas of combat operations, (5) aggressiveness from the local population, (6) distress among the local population, (7) being exposed to injured people, (8) being exposed to dead people, (9) witnessing atrocities against civilians and (10) experiencing lack of timely reinforcement.

### 2.4. Procedure

Data for the pre-established PTSD trajectories were collected 5–6 weeks before deployment, during deployment (approximately 3 months into the deployment, at the base or in the Afghan airports), 1–3 weeks after homecoming at homecoming meetings, 2 months after homecoming, 7 months after homecoming and 2.5 years after homecoming. Written informed consent was obtained from all the participants after they had been provided with information about the study. They were assured of the anonymity of their responses, that data would only be used for research purposes and that no military leaders would gain access to the data. The study was approved by the Danish Data Protection Agency (Copenhagen, Denmark). Detailed data collection procedures for the PTSD trajectories are described elsewhere (Andersen et al., 2014).

Response rates at each assessment were: at pre-deployment 81%, during deployment 79%, at homecoming 74%, 2 months after homecoming 45%, 7 months after homecoming 42% and 2.5 years after homecoming 78%. A general analysis of the response rates showed no significant differences between soldiers who answered before, at homecoming and 2.5 years after homecoming (Danish Defence, 2013). Further, previous analyses have found no differences between the latent growth mixture modelling (LGMM) model established based on the 561 individuals included here and one including only those responding four or more times (Andersen et al., 2014). Further descriptive information on the measurements at pre-deployment, after homecoming and post-deployment from this study is shown in Table 1.

**Table 1.** Measurements at pre-deployment, 1–3 weeks after homecoming and 2.5 years after homecoming.

	*n*	*M*	*SD*
Measurements before deployment			
Dissociation	579	10.84	10.82
PTSD (PCL-C)	602	22.54	7.08
Previous trauma	596	10.57	9.80
Neuroticism	583	29.77	6.23
Extraversion	579	43.67	6.24
Openness	579	37.64	6.17
Agreeableness	582	40.19	6.09
Conscientiousness	576	44.27	6.10
Measurements 1–3 weeks after homecoming			
Perceived war-zone stress	520	20.29	5.09
PTSD (PCL-C)	550	21.24	7.11
Measurements 2.5 years after homecoming			
PTSD (PCL-C)	533	27.50	11.79

*n*, sample; *M*, mean score; *SD*, standard deviation; PTSD, post-traumatic stress disorder; PCL-C, PTSD Checklist – Civilian version.

### 2.5. Data analysis

The dissociation data displayed marked positive skew and a logarithmic transformation log_10_(*X* + 1) was performed so that robust parametric tests could be applied for analysis, as suggested by Howell (2010). After log-transformation the data were normally distributed.

**Figure 1.**
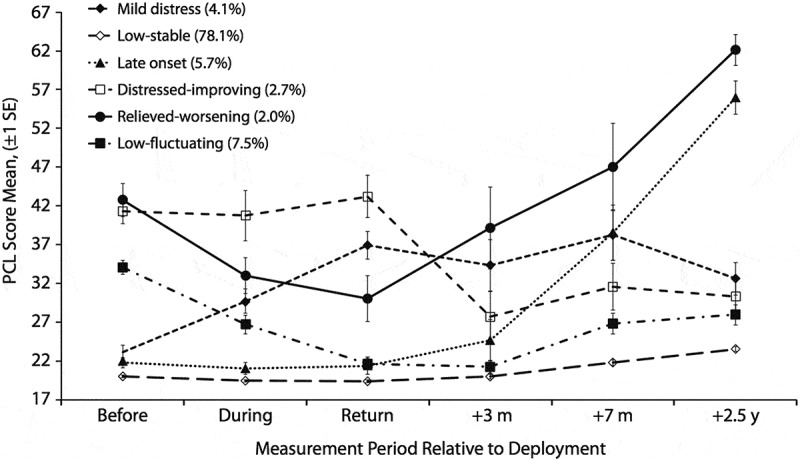
Developmental trajectories of post-traumatic stress disorder (PTSD) symptoms at six time points before, during and after deployment (N=561)  (Andersen et al., 2014). PCL, PTSD Checklist; *SE*, standard error.

The analyses were performed in three stages. For the first analysis, we used the post-traumatic stress trajectories previously identified by the application of LGMM (Andersen et al., 2014). The six trajectories were established at six time points before, during and after deployment over a period of 3 years, as shown in Figure 1. These consisted of a low–stable group with low symptom levels across all six measurement occasions, which encompassed the majority of the sample (*n* = 438, 78.1%). The other five groups had fluctuating symptom levels before, during and after deployment and can be described as: the low–fluctuating group (*n* = 42, 7.5%) with mild symptoms before deployment, symptom decrease during deployment and mild increase after homecoming; the late-onset group (*n* = 32, 5.7%) with low stable symptoms until 3 months after return, followed by a high symptom increase; the mild-distress group (*n* = 23, 4.1%) with low initial symptoms which increased to subclinical levels during and after deployment; the distressed–improving group (*n* = 15, 2.7%) with mild symptom increase during deployment followed by symptom relief at homecoming; and the relieved–worsening group (*n* = 11, 2.0%) with symptom relief during deployment followed by drastic symptom increase ongoing through 2.5 years after homecoming. To estimate the association between pre-deployment dissociation and PTSD trajectory membership, we used general linear model (GLM) analysis instead of logistic regression. The reason for this is that logistic regression maximizes overall classification accuracy. Hence, it automatically favours large groups and disfavours small groups with regard to correct classification (Finch & Schneider, 2006). Since the population of each PTSD trajectory varies considerably, from 2% to 78.1%, the classification accuracy for each trajectory/level in a logistic regression model will also differ considerably. As a result, the classification accuracy for small trajectories will be unreliable. Therefore, univariate GLM is used as it is a more robust model and is not affected by this issue.

For the second analysis, we used Pearson correlation to assess the association between the Big Five personality traits and dissociation. We also included a previous history of trauma (total TLEQ score) in the correlations.

For the third analysis, we used a two-step linear regression model to identify the pre-deployment personality variables that could be risk factors for post-deployment PTSD symptoms (measured by the PCL 2.5 years after homecoming). In step 1, the model included all predictor variables of interest to make sure that we accounted for any shared variability. Here, we also included the TLEQ to control for any variability related to previous trauma, the Danger–Injury Exposure Scale to control for any variability related to perceived war-zone stress, and the cross-product between neuroticism and dissociation to test for interaction effects on the outcome. We used the ‘enter’ method: all the variables are entered into the equation at the same time and the most non-significant variable is removed each time. The final analysis provides the best-fit model in step 2, which includes the predictor variables: TLEQ, neuroticism and dissociation.

## 3. Results

### 3.1. Association between pre-deployment dissociation and PTSD symptom trajectories

GLM analyses indicated statistically significant mean differences in pre-deployment dissociation scores for the six PTSD trajectories. A univariate analysis of variance showed a statistically significant difference between groups (*F*(5,525) = 14.27, *p *< 0.001, *η*^2^ = 0.120). Thus, the null hypothesis of no differences in dissociation between trajectories was rejected, and 12% of the variance in dissociation was accounted for by trajectory membership. The statistically significant effect corresponds to a moderate to large effect size based on Cohen’s guidelines (Cohen, 1992). Bonferroni post hoc tests, as shown in Table 2, evaluated the nature of the significant mean differences in pre-deployment dissociation for the PTSD trajectories. These significant mean differences indicated specifically that the relieved–worsening trajectory had higher pre-deployment dissociation levels compared to the low–stable trajectory. Further, the low–fluctuating trajectory had higher pre-deployment dissociation levels compared to the low–stable, mild-distress and late-onset trajectories. Lastly, the distressed–improving trajectory had higher pre-deployment dissociation levels compared to the low–stable trajectory.

**Table 2.** Significant mean differences in dissociation for six post-traumatic stress disorder (PTSD) trajectories (*n* = 561).

Trajectory	M dissociation	PCL-C	Relieved–worsening	Low–fluctuating	Distressed–improving	Low–stable	Mild distress	Late onset
Relieved–worsening (*n* = 7)	21.49	42.73	–			12.54*		
						[1.1, 24.0]		
Low–fluctuating (*n* = 38)	20.77	34.05		–		11.82*	8.63*	7.80*
						[6.7, 16.9]	[0.6, 16.7]	[0.4, 15.2]
Distressed–improving (*n* = 15)	19.97	41.27			–	11.02* [3.1, 18.9]		
Low–stable (*n* = 420)	8.95	20.01	−12.54*	−11.82*	−11.02*	–		
			[−24.0, −1.1]	[−16.9, −6.7]	[−18.9, −3.1]			
Mild distress (*n* = 22)	12.13	23.13		−8.63*			–	
				[−16.7, −0.6]				
Late onset (*n* = 29)	12.97	21.78		−7.80*				–
				[−15.2, −0.4]				

**Figure 2.**
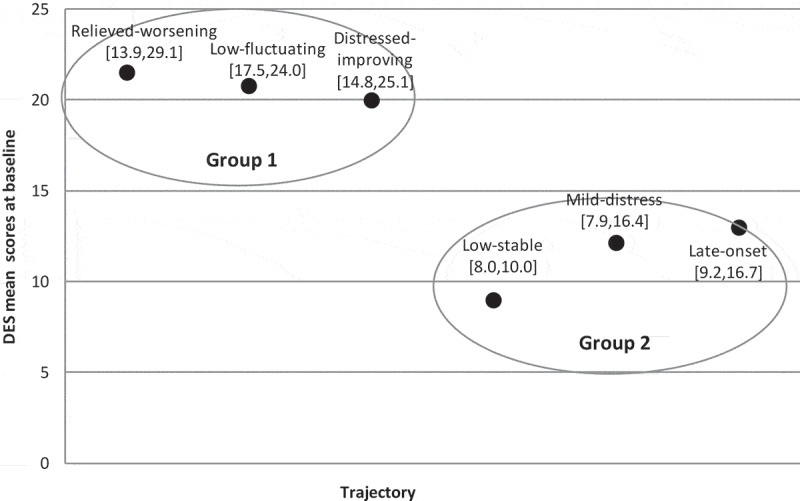
Two distinct groups of post-traumatic stress disorder trajectories in relation to differences in pre-deployment dissociation, 95% confidence intervals for the mean. DES, Dissociative Experience Scale.

The right upper quadrant and the left lower quadrant show significant mean differences in dissociation, between specific combinations of trajectories. The left upper blank quadrant and the right lower blank quadrant show that there are no significant mean differences in dissociation between these specific trajectories, which explains why these trajectories form the two groups illustrated in Figure 2.

*M*, mean score in pre-deployment dissociation; PCL-C, PTSD Checklist – Civilian version symptom scores measured at pre-deployment.

*Significant mean differences in dissociation at *p* < 0.05; 95% confidence interval in brackets.

Based on the above significant differences, two main groups of PTSD trajectories emerged, as illustrated in Figure 2. The first group consisted of the relieved–worsening, low–fluctuating and distressed–improving trajectories, and exhibited high dissociation scores at pre-deployment. The second group consisted of the low–stable, mild-distress and late-onset trajectories, and exhibited low dissociation scores at pre-deployment.

### 3.2. Relationship between the Big Five model of personality, pre-deployment dissociation and previous history of trauma

Pearson correlations showed a moderate positive correlation between dissociation and neuroticism at pre-deployment (*r* = 0.31, *n* = 555, *p* < 0.001) and a weak negative correlation between dissociation and conscientiousness (*r* = −0.22, *n* = 548, *p* < 0.001). There were no significant correlations between dissociation at pre-deployment and the other personality constructs: extraversion, openness and agreeableness. We also found a weak positive correlation between dissociation and the total TLEQ score (*r* = 0.22, *n* = 565, *p* < 0.001). The correlation between neuroticism and the total TLEQ score was not significant.

### 3.3. Personality and dissociation as risk factors for post-deployment PTSD measured 2.5 years after homecoming

We used linear regression to determine the factors associated with post-deployment PTSD. As indicated in Table 3, step 1 includes all the variables and these explained 16.4% of the variance (*F*(9,394) = 8.61, *p* < 0.001, *R*^2^ = 0.164). In step 2, all the non-significant variables were removed and the model indicated that three variables: previous trauma, dissociation and neuroticism, had a significant effect on post-deployment PTSD. In this model, the three variables explained 15.8% of the variance (*F*(3,448) = 27.95, *p* < 0.001, *R*^2^ = 0.158), and standardized coefficients are shown in Table 3. We controlled the confounding effect of demographic factors such as age, gender, civil status, years in service and education in linear regression. These variables were non-significant.

**Table 3.** Linear regression: dissociation, the Big Five, previous trauma and war-zone stress association with development of post-deployment post-traumatic stress disorder (PTSD) 2.5 years after homecoming (*n* = 533).

Variable	*β*	*SE*	*t*	*p*	*R*^2^
Step 1					
Danger–Injury	0.02	0.12	0.31	0.76	0.164
Previous trauma	0.33	0.06	6.55	0.00*	
Neuroticism	0.24	0.25	1.85	0.07	
Extraversion	0.07	0.10	1.23	0.22	
Openness	0.01	0.10	0.12	0.90	
Agreeableness	0.03	0.10	0.62	0.54	
Conscientiousness	−0.03	0.11	−0.48	0.63	
Dissociation	0.35	7.29	1.59	0.11	
Neuroticism Dissociation	−0.32	0.24	−1.14	0.26	
Step 2					
Previous trauma	0.34	0.05	7.64	0.00*	0.158
Neuroticism	0.09	0.09	2.06	0.04*	
Dissociation	0.11	1.54	2.28	0.02*	

In the variable column: perceived war-zone stress (Danger–Injury Exposure Scale) was measured at homecoming. All other variables were measured at pre-deployment. Step 1 includes all predictor variables in the model. The final analysis provides the best-fit model in step 2, which includes the predictor variables that make a significant contribution.

*β*, standardized coefficient; *SE*, standard error.

*p < 0.05.

## 4. Discussion

The aim of the study was to estimate the influence of pre-deployment dissociation on the development of PTSD 2.5 years after homecoming using previously identified PTSD trajectories and end-point PTSD symptoms. The findings of this study show significant differences in pre-deployment dissociation levels for the six trajectories of PTSD symptoms.

The results of the previous USPER study from this cohort suggested that higher levels of pre-deployment emotional problems and pre-deployment trauma were predictors for inclusion in the five groups that had fluctuating symptom levels compared to the resilient group with low–stable symptom levels (Andersen et al., 2014). Owing to dissociation’s association with history of previous trauma (Morgan et al., 2001), high dissociation levels in the five groups with fluctuating symptom levels would have been expected. However, we observed a specific pattern of associations that differentiated pre-deployment dissociation into two groups of PTSD trajectories. Group 1 consisted of the relieved–worsening, low–fluctuating and distressed–improving trajectories and was characterized by having high pre-deployment dissociation. Group 2 consisted of the low–stable, mild-distress and late-onset trajectories and was characterized by having low pre-deployment dissociation.

The trajectories in group 1 exhibited higher pre-deployment PCL-C scores than the trajectories with low dissociation in group 2. This is in line with previous evidence indicating that dissociation is strongly related to the presence and severity of PTSD symptoms (Dalenberg & Carlson, 2012). The results also suggest that the trajectories with higher pre-deployment dissociation could be at greater risk for developing pathological dissociation, with a subsequent increase in PTSD symptoms. However, this idea is compromised by the fact that those in the late-onset trajectory were in the ‘low-dissociation’ group. Hence, other pre- and post-deployment risk factors may be at play. Indeed, according to a previous study of this sample, pre-deployment emotional problems and exposure to traumatic situations after homecoming increased the risk of the development of PTSD in the late-onset group (Andersen et al., 2014).

Furthermore, in our study, the average pre-deployment dissociation scores were ≤ 21.49, indicating, on average, non-pathological dissociation. However, lower scores have been identified in clinical populations (Brand, Myrick, Sar, & Lanius, 2011), and one must be careful in interpreting the scores of this cohort because dissociation alone may not necessarily be indicative of pathology, but it may contribute to psychopathology if combined with other traits and factors. Hence, the further contribution of personality traits from the Big Five model and previous history of trauma was explored in this study.

We found a positive association between previous history of trauma and pre-deployment dissociation, suggesting that previous life adversities could have reduced the individual’s ability to regulate affect, resulting in dissociative symptoms, as Van Der Hart and Steele (2013) indicated in their study. Further evidence that pre-existing dissociation symptoms are linked to early trauma history is found in studies related to military training. For instance, having a history of previous trauma significantly influenced the degree of dissociative symptoms prior to and in response to stress during survival training among general infantry soldiers and Special Forces soldiers in the US Army (Morgan et al., 2001). Similarly, women in the US Navy with previous exposure to potentially traumatic life events reported dissociation symptoms at baseline (before survival training), which increased during intense survival military training (Dimoulas et al., 2007).

Furthermore, our findings showed a positive association between dissociation and neuroticism. Although causality cannot be presumed from the analysis, previous studies suggest that it is possible that the increased emotionality that characterizes neuroticism may enhance dissociative experiences (Spindler & Elklit, 2003). As a result, repeated use of dissociation may become embedded in a coping mechanism, leading to pathology (Ginzburg, Solomon, Dekel, & Bleich, 2006; van der Hart & Steele, 2013) and perpetuating PTSD symptomatology (Foa & Hearst-Ikeda, 1996). We also found a negative association between dissociation and conscientiousness. Conscientiousness refers to the ability to consider others in decision-making, impulse control and goal-oriented behaviours. While conscientiousness signifies engagement with the external world, dissociation represents an experiential detachment and a sense of estrangement from self or others (Cardeña & Carlson, 2011). The lack of associations between dissociation and other personality constructs in this study is in line with previous evidence (Kwapil et al., 2002; Spindler & Elklit, 2003).

Finally, when considering multiple variables and traits, our study showed that previous history of trauma, pre-deployment dissociation and pre-deployment neuroticism were significant independent predictors of post-deployment PTSD, with previous history of trauma being the strongest predictor. This is consistent with previous studies showing history of trauma as a risk factor in combat-related PTSD (Xue et al., 2015). There is also evidence of a negative influence of neuroticism in coping with trauma in combat soldiers (Engelhard & van den Hout, 2007). In this regard, it is likely that individuals high on the neuroticism trait display problems with emotional regulation that may enhance dissociative experiences or, in combination with dissociation, add to vulnerability. In relation to the latter, we found no interaction effects of neuroticism and dissociation on post-deployment PTSD; nevertheless, the results support the independent contribution of multiple factors.

The present study has several limitations. Owing to selection practices in the military, individuals with severe psychopathology may have been excluded. In this regard, we do not know whether selection could have had an influence in decreasing variance in dissociation. Moreover, without a non-military control group causal conclusions on the relationship between dissociation and neuroticism cannot be drawn. Other selection biases may be due to attrition. However, a general analysis of the response rates showed no significant differences between soldiers who answered before homecoming, at homecoming and 2.5 years after homecoming (Andersen et al., 2014). There may be potential response bias due to self-report measures. For instance, social desirability is a common concern in personality research; however, this issue was minimized by ensuring anonymity and confidentiality.

Despite the limitations, the foremost strength of this study is the prospective longitudinal design, handing us the possibility to examine the link between pre-deployment dissociation and six PTSD symptom trajectories as well as end-point PTSD symptoms. The findings of our study contribute to the literature by revealing two unique groups of trajectories based on differences in pre-deployment dissociation. In addition, it highlights the importance of examining the influence of multiple personality traits as risk factors for PTSD. Further research should focus on the combined effect of several predispositions and traits in the development of PTSD, and should include patient populations with higher symptom loads in military as well as civilian populations.

## 5. Conclusion

Our findings highlight the influence of pre-deployment dissociation in post-deployment PTSD, and underscore the importance of co-occurring processes. Although we cannot draw causal conclusions, previous life adversities, even if not traumatic, may trigger a predisposition to dissociate. Neuroticism may also add to the combined vulnerability, owing to the negative emotionality associated with it. The findings of this study have clinical and practical significance. In the clinical setting, close attention must be given to the individual traits of neuroticism and dissociation because of their associations with PTSD. Individuals with high neuroticism may be less able to regulate emotions and may be more prone to use dissociative strategies, exacerbating and perpetuating PTSD symptomatology. The study underscores the importance of pre-deployment dissociation and personality traits in pre-deployment assessments in the military setting and other law enforcement areas, as they may play an important role in military performance, differential adaptation to stress and subsequent psychological well-being.
